# The 482Ser of *PPARGC1A* and 12Pro of *PPARG2* Alleles Are Associated with Reduction of Metabolic Risk Factors Even Obesity in a Mexican-Mestizo Population

**DOI:** 10.1155/2015/285491

**Published:** 2015-06-22

**Authors:** Mónica Vázquez-Del Mercado, Milton-Omar Guzmán-Ornelas, Fernanda-Isadora Corona Meraz, Clara-Patricia Ríos-Ibarra, Eduardo-Alejandro Reyes-Serratos, Jorge Castro-Albarran, Sandra-Luz Ruíz-Quezada, Rosa-Elena Navarro-Hernández

**Affiliations:** ^1^Instituto de Investigación en Reumatología y del Sistema Musculo Esquelético, Centro Universitario de Ciencias de la Salud, Universidad de Guadalajara, Sierra Mojada No. 950, Colonia Independencia, 44340 Guadalajara, JAL, Mexico; ^2^Centro Universitario de Ciencias de la Salud, Universidad de Guadalajara, Sierra Mojada No. 950, Colonia Independencia, 44340 Guadalajara, JAL, Mexico; ^3^Servicio de Reumatología, Hospital Civil “Dr. Juan I. Menchaca”, Salvador Quevedo y Zubieta No. 750, Colonia Independencia, 44340 Guadalajara, JAL, Mexico; ^4^UDG-CA-701, Grupo de Investigación Inmunometabolismo en Enfermedades Emergentes (GIIEE), Centro Universitario de Ciencias de la Salud, Universidad de Guadalajara, Sierra Mojada No. 950, Colonia Independencia, 44340 Guadalajara, JAL, Mexico; ^5^Tecnológico de Monterrey, Campus Guadalajara, Avenida General Ramón Corona No. 2514, Colonia Nuevo México, 45201 Zapopan, JAL, Mexico; ^6^Departamento de Farmacobiología, Centro Universitario de Ciencias Exactas e Ingenierías, Universidad de Guadalajara, Boulevard Marcelino García Barragán 1421, 44430 Guadalajara, JAL, Mexico; ^7^HMIELM, Secretaria de Salud Jalisco, Avenida Constituyentes 1075, Colonia Moderna, 44190 Guadalajara, JAL, Mexico

## Abstract

The aim of this study was to investigate the relationship between functional polymorphisms Gly482Ser in *PPARGC1A* and Pro12Ala in *PPARG2* with the presence of obesity and metabolic risk factors. We included 375 individuals characterized as Mexican-Mestizos and classified by the body mass index (BMI). Body dimensions and distribution of body fat were measured. The HOMA-IR and adiposity indexes were calculated. Adipokines and metabolic profile quantification were performed by ELISA and routine methods. Genetic polymorphisms were determined by polymerase chain reaction restriction fragment length polymorphism analysis. A difference between obese and nonobese subjects in polymorphism *PPARGC1A* distribution was observed. Among obese individuals, carriers of genotype 482Gly/Gly were observed to have decreased body fat, BMI, and body fat ratio versus 482Ser/Ser carriers and increased resistin and leptin levels in carriers Gly+ phenotype versus Gly− phenotype. Subjects with *PPARG2* Ala− phenotype (genotype 12Pro/Pro) showed a decreased HOMA-IR index versus individuals with Ala+ phenotype (genotypes 12Pro/Ala plus 12Ala/Ala). We propose that, in obese Mexican-Mestizos, the combination of alleles 482Ser in *PPARGC1A* and 12Pro in *PPARG2* represents a reduced metabolic risk profile, even when the adiposity indexes are increased.

## 1. Introduction

Obesity is the result of a positive energetic balance that has been maintained for a long period of time. It is characterized by an excessive and continuous fat deposit in adipose tissue and is one of the most common metabolic disorders [1]. Obesity is considered a major factor that triggers metabolic risk and the development of secondary chronic illness [2].

The susceptibility of a subject to develop obesity will depend on different factors such as the repertoire of individual variations in an ensemble of relevant genes, their history of exposure to environmental risk factors, and the interaction between the lifestyle and metabolism, which is also modulated by the genetic components [3–5].

The identification of diverse molecular mechanisms related to energy metabolism has allowed the definition of strategies for searching genes implied in obesity. Among candidate genes that have been studied,* PPARGC1A* (peroxisome proliferator-activated receptor gamma, coactivator 1 alpha) and* PPARG2* (peroxisome proliferator-activated receptor gamma 2) follow the same metabolic pathway [1, 2, 6].

In human beings,* PPARGC1A* gene is located on chromosome 4p15.1 and codes for a 798 amino-acid protein (PGC-1*α*), which is highly expressed on tissues where mitochondria are abundant and oxidative metabolism is activated, such as heart and skeletal muscle. It also plays a role in the pathogenesis of obesity by favoring the preadipocytes differentiation into adipocytes and by regulating the energy balance [7]. In* PPARGC1A,* a polymorphism that consists in a transition at codon 482 (rs8192678) was associated with chronic diseases [8–13].

The* PPARG2* gene codes for PPAR*γ*2 protein that is mainly expressed in adipose tissue and is the central engine in adipocyte differentiation. When activated by an agonist ligand in preadipocytes, a differentiation program is stimulated; in turn, morphological changes, lipid accumulation, and distinctive adipocyte gene expression take place [14–17].

*PPARG2 *is located on chromosome 3 and its polymorphism Pro12Ala, rs1801282 is located in exon B.* In vitro* analyses on its functionality have shown controversial results regarding the 12Ala allele and the reduction in the transcriptional activity of* PPARG2*, while the polymorphism's involvement in T2DM is reported in several studies [13, 18–20].

In this context, it has been shown that obesity is due mainly to an increase in adiposity, where many functional genes are altered; among them are* PPARG2* and its coactivator* PPARGC1A*. In adipose tissue, the expression of these genes is modified unfavorably reflecting altered adipogenesis and glucose metabolism. The Gly482Ser and Pro12Ala polymorphisms have been related to the development and severity of metabolic diseases, which suggests functional modification of the protein. Hence, the main objective of this study was to identify the relationship between polymorphisms Gly482Ser in* PPARGC1A* and Pro12Ala in* PPARG2* and metabolic risk factors in normal, preobese, and obese Western Mexican-Mestizo population.

## 2. Materials and Methods

### 2.1. Study Design

A cross sectional study was performed. Each selected subject was requested to sign an informed written consent approved by the IRB committee of Centro Universitario de Ciencias de la Salud, Universidad de Guadalajara. A medical doctor confirmed a clinical healthy condition and stable weight for the past three months in every subject. Individuals that presented acute or chronic diseases, infections, and T2DM were not included.

The selected subjects were older than 18 years old, Mexican-Mestizos of Western of Mexico [21]. They were classified based on two criteria as follows: (1) normal (BMI: 18.5–24.9 kg/m^2^), preobese (BMI: 25–29.9 kg/m^2^), and obese (BMI > 30.0 kg/m^2^) and (2) with obesity (BMI > 30.0 kg/m^2^) and without obesity (BMI: 18.5–29.9 kg/m^2^) [22].

### 2.2. Data Collection Methods

The study group constituted of 375 individuals. The clinical and nutritional evaluation data were obtained through questionnaires and general physical examinations.

### 2.3. Body Dimensions and Distribution of Body Fat Storage Measurements Included

Height (Seca GmbH & Co KG. Hamburg, Germany, stadiometer to accurateness of 1.0 mm), body weight, and body composition (measured with the electric bioimpedance method to the precision of 0.1 kg by TANITA's TBF304 system; Tokyo, Japan) [23, 24], waist and hip circumferences [25] with GULICK fiberglass retractable measurement tape (North Coast Medical Inc., Gilroy, CA). The obesity and adiposity indexes were calculated according to anthropometric indicators measurement guides: body mass index (kg/m^2^) = weight (kg)/height^2^ (m) [26]; body fat ratio = total body fat mass (kg)/height^2^ (m) [27]; waist hip/ratio (WHR) = waist circumference (WC, cm)/hip circumference (cm) [28]; waist height ratio (WthR) = waist circumference (cm)/height (cm) [29]; conicity index = WC (cm)/0.109*√*(weight (kg)/height (cm)) [30]; abdominal volume index (*L*) = (WC^2^ + (0.7 cm) (WC − HC)^2^)/1000 [28]; total adipose area (cm^2^) = WC^2^/4*π* [29]. The homeostasis model assessment-insulin resistance (HOMA-IR) [31] index was calculated.

### 2.4. Adipokines and Metabolic Markers Profile Quantification

Enzymatic and immunoturbidimetry assays (Randox Laboratories; 55 Diamond Road, Crumlin Co. Antrim, Northern Ireland, UK) were performed to quantify basal serum glucose, and lipid profile (i.e., triglycerides, total cholesterol, high and low density lipoproteins cholesterol (HDLc and LDLc, resp.), apolipoprotein A-1, and apolipoprotein B). Very low density lipoprotein cholesterol (VLDLc) was quantified using the Friedewald formula [32].

ELISA was used to quantify basal serum insulin levels (detection limit: 0.399 *μ*IU/mL), adiponectin (detection limit: 0.019 ng/mL), leptin (detection limit: 0.42 ng/mL); (ALPCO Diagnostics 26-G Keewaydin Drive, Salem NH 03079) and resistin levels (detection limit: 0.4 ng/mL; Enzo Life Sciences, Inc.; New York, NY, USA).

### 2.5. Genotyping Techniques

To identify genetic polymorphisms, genomic DNA was isolated from total blood by the modified Miller's method [26] and stored at −20°C until genotyping.

The collected DNA underwent polymerase chain reaction- (PCR-) restriction fragment length polymorphism (RFLP) analysis; primers were* PPARGC1A* polymorphism F: 5′-TGCTACCTGAGAGAGACTTTG-3′, R: 5′-CTTTCATCTTCGCTGTCATC-3′,* PPARG2: F: 5*′*-*CAGTGTGGCAATATTTTCCCTGTA-*3*′, and R: 5′GTATCAGTGAAGGAATCGCTTTC**C**-3′ (this primer containing one nucleotide mismatch (underlined), which made it possible to use the restriction enzyme* Msp*I).

The PCR was performed with a 25 *μ*L reaction mixture (100 ng of DNA, 2 nM of each primer, 2.5 mM of each dNTP, 1.5 mM of MgCl_2_, 0.25 U Taq polymerase, and 1X PCR buffer, (Invitrogen Life Technologies; Thermo Fisher Scientific Inc.)). PCR was performed with an initial melting step of 4 min at 94°C, followed by 35 cycles of 30 s at 94°C, 30 s at 56/60°C (Gly482Ser and Pro12Ala polymorphisms, resp.), 30 s at 72°C, and a final elongation step of 4 min at 72°C.

To determine the changes in specific alleles of each individual, PCR products were submitted to a digestive process using the* Msp*I enzyme (New England BioLabs Inc.; 240 Country Road, Ipswich, MA).

The obtained restriction fragments (149 and 111 bp for Gly allele, and 260 bp for Ser allele,/326 and 25 bp for Pro allele, and 351 bp for Ala allele) were observed in 4% agarose gels stained with ethidium bromide and photographed under UV light. To ensure the accuracy of genotype data, we used internal controls and repetitive experiments; that is, all samples were repeated at random to verify the reproducibility, with positive controls in each experiment. The genotyping success rate was 100%.

### 2.6. Statistical Analysis

The statistical analysis was made with the software SPSS v21 (IBM Inc., Chicago, IL, USA) and GraphPad Prism v6.01 (©2014 Inc. 2236, Avenida de la Playa, La Jolla, CA 92037). Results are given as mean ± SD or percentages.

The clinical and laboratory characteristics of the study group were performed with the unpaired Student's *t*-test, and to compare quantitative data in the three groups studied (normal, preobese and obese), a one-way ANOVA and post hoc Tukey's test were used. Data from serum concentrations of metabolic biomarkers with body adipose tissue variables were subjected to Pearson or Spearman correlation tests.

The test for Hardy-Weinberg equilibrium [33] for individual loci was performed for deviation. Contingency tables (2 × 2, 3 × 2 and 3 × 3) with *χ* ^2^ trend test or Fisher exact test, depending on the case, were used for testing the differences of genotype distributions and allele frequencies between all subgroups. Two genetic models were used for these analyses: (i) the dominant model where each SNP was modeled categorically and separated into three categories, one for each genotype, and (ii) the phenotype model, where each polymorphism was modeled into two categories, with two genotypes combined into one category, choosing one genotype as the reference group, (i.e., polymorphism* PPARGC1A*: phenotype Ser+ (Genotypes 482Ser/Ser plus 482Gly/Ser) and, phenotype Ser− (genotype 482Gly/Gly); polymorphism* PPARG2*: phenotype A− (genotype 12Pro/Pro) and phenotype Ala+ (genotypes 12Pro/Ala plus 12Ala/Ala)).

Genotype and phenotype intergroup comparisons by means of all variables were performed using one-way ANOVA with Tukey's test and, Student *t*-test for normally distributed traits, analysis of ranks for traits with nonnormal distributions with Kruskal-Wallis and Mann-Whitney *U* test, as appropriate. A two-tailed *P*-value less than 0.05 was considered statistically significant.

## 3. Results

### 3.1. Clinical and Demographic Characteristics of the Whole Group

The cohort was composed of 375 Mexican-Mestizos ($\bar{x}=37.8\pm 13.5$ years old) of which 133 were men ($\bar{x}=36.2\pm 13.3$ years old) and 242 were women ($\bar{x}=38.6\pm 13.6$ years old). Twenty-one percent of obesity and 59% of overweight frequencies were noted on the whole group. Abdominal obesity frequencies were 3.2, 32.9, and 74.7% with dyslipidemias of 16.8, 32.9, and 31.3% in normal range, preobese and obese individuals, respectively. The adiposity measurements and metabolic markers profile according to BMI and correlations in the whole group studied are shown in Tables 1 and 2. A positive correlation of glucose and insulin levels, HOMA-IR, and lipid profile was seen along accumulation of abdominal fat and an increase in adiposity indexes, except HDLc and apolipoprotein A-1. These two lipoproteins correlated negatively with abdominal obesity indicators (Table 2).

### 3.2. Distribution of Polymorphisms of* PPARGC1A *and* PPARG2* in the Whole Group

In our cohort, the observed and expected frequencies of polymorphisms seen in individuals with BMI in normal range are according with the Hardy-Weinberg principle.

When we analyzed the Gly482Ser polymorphism of* PPARGC1A* we observed a similar distribution of the genotypes in individuals classified according to the WHO criteria on BMI.

The analysis of polymorphism of* PPARG2* showed a difference in genotypic distribution in individuals classified according to the WHO criteria on BMI. We observed a difference in genotype and phenotype distribution in subjects without obesity when compared to individuals designated as obese (Table 3).

### 3.3. Body Distribution and Clinical Measurements in Normal, Preobese and Obese BMI Subjects by Genotypes and Phenotypes of* PPARGC1A* and* PPARG2*

Body fat distribution characteristics of the group are shown according to genotypes and phenotypes in normal range (Table 4(a)), preobese (Table 4(b)) and obese BMI subjects (Table 4(c)).

A difference in the level of glucose was found in the normal range subjects bearing genotype Gly/Ser* versus* Ser/Ser, while a difference in triglycerides level was observed in the preobese subjects when comparing Ser− versus Ser+ phenotypes, and in HDLc levels for Ala− versus Ala+ phenotypes (Table 4(b)).

On the other hand, in obese individuals, we observed an increase in metabolic markers (glucose, insulin, and LDLc), increased HOMA-IR index, and low levels of total adiponectin in Ala+ versus Ala− phenotype carriers.

In carriers Ser+ versus Ser− phenotypes, a decrease of triglycerides and apolipoprotein B and increase of apolipoprotein A-1 levels were observed. Body dimensions (body weight, total body fat mass, and hip circumference), adiposity indexes (BMI, body fat ratio, and WHR), and conicity index were increased in carriers of polymorphic alleles 482Ser and 12Ala, respectively (Table 4(c)).

### 3.4. Metabolic, Adiposity Markers, Adipokines in the Whole Group according to Genotypes and Phenotypes of* PPARGC1A* and* PPARG2*

**Figure 1:**
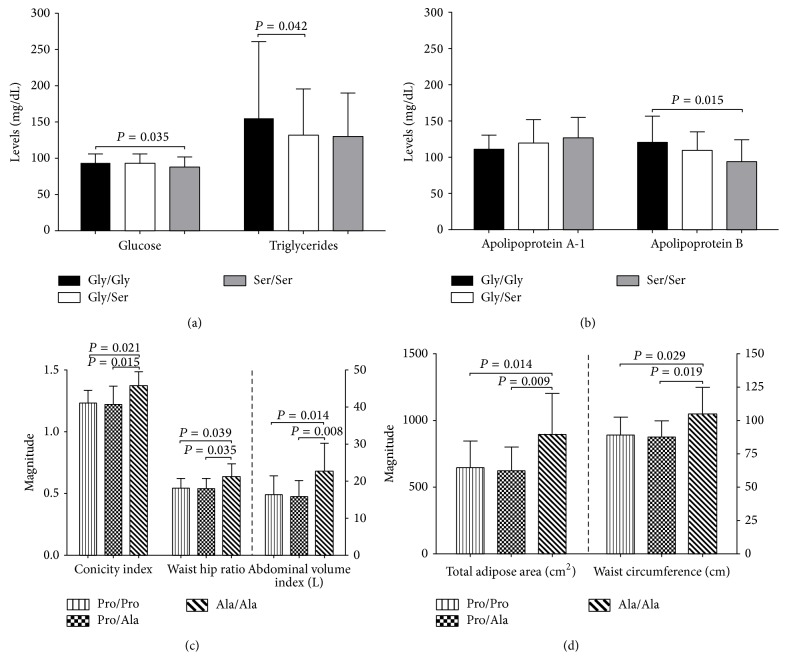
Metabolic markers, body dimensions and distribution of body fat storage measurements in study group by genotypes. (a) And (b)* PPARGC1A* Gly482Ser polymorphism; (c) and (d) Pro12Ala in* PPARG2*. One-way ANOVA test with Tukey's post hoc test. Data are means ± SD. *n* = 375.

When comparing subjects carrying polymorphic genotype 482Ser/Ser versus the wild-type genotype (i.e.: 482Gly/Gly), a decrease in glucose and apolipoprotein B levels was observed (Figures 1(a) and 1(b)). In the carriers of 12Ala/Ala polymorphic genotype, increase in dimensions and abdominal fat accumulation was observed when compared to carriers of the wild type genotype 12Pro/Pro and heterozygotes 12Pro/Ala (Figures 1(c) and 1(d)).

The most relevant data were obtained in obese individuals (i.e.: BMI > 30 kg/m^2^).

**Figure 2:**
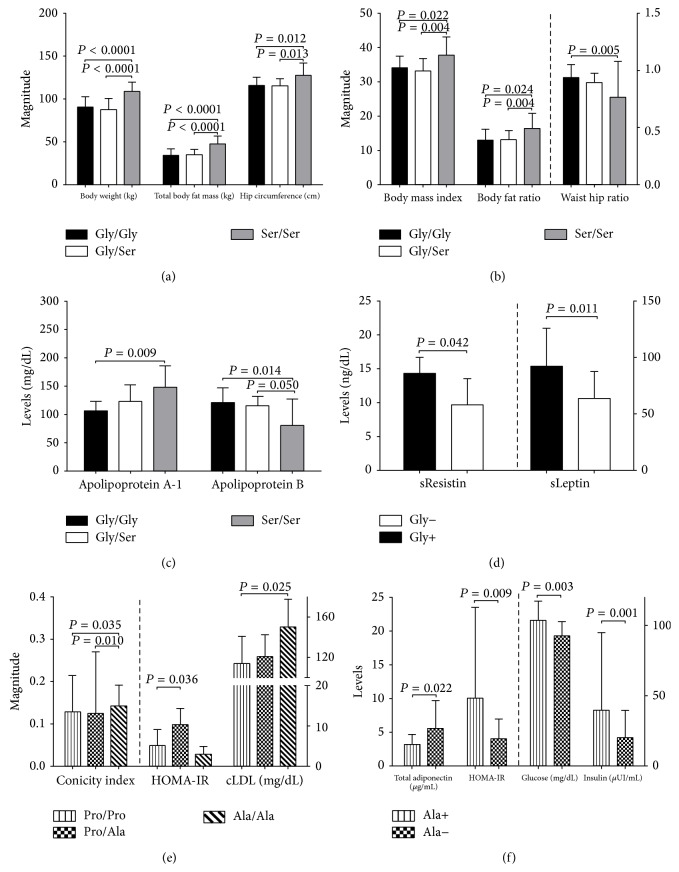
Metabolic markers, adipokines, and body dimensions and distribution of body fat storage measurements in obese subjects by genotypes and phenotypes. (a), (b), (c) and (d)* PPARGC1A* Gly482Ser polymorphism; (e) and (f) Pro12Ala in* PPARG2*. One way ANOVA test with Tukey's post hoc test, and ^*^Student's  *t*-test. Polymorphism* PPARGC1A*: phenotype Gly− (Genotype 482Ser/Ser) and, phenotype Gly+ (genotypes 482Gly/Ser plus 482Gly/Gly). Polymorphism* PPARG2*: phenotype Ala+ (genotypes 12Pro/Ala plus 12Ala/Ala) and phenotype Ala− (genotype 12Pro/Pro). Data are means ± SD. *n* = 78.

- Increase in body weight, total body fat mass, hip circumference, BMI, and body fat ratio, was seen in subjects carrying genotype Ser/Ser in comparison to carriers of genotypes Gly/Gly and Gly/Ser (Figures 2(a), and 2(b), resp.), as well as a parallel decrease in apolipoprotein B levels and WHR in carriers of Ser/Ser genotype individuals (Figures 2(c) and 2(b), resp.).
- Increase in LDLc levels in carriers of genotype 12Ala/Ala was seen as compared to carriers of genotype 12Pro/Pro. Also, an increase in the magnitude of conicity index in carriers of genotypes 12Pro/Pro and 12Pro/Ala was observed, along with increase in the HOMA-IR index magnitude when compared to 12Pro/Pro subjects (Figure 2(e)).
- Decrease in resistin and leptin levels in individuals Gly− when compared to Gly+ phenotypes (Figure 2(d)); whereas subjects with* PPARG2*'s Ala− phenotype showed a decrease in HOMA-IR index, glucose, and insulin's basal levels and an increase in total adiponectin serum levels* versus* carriers of the Ala+ phenotype (Figure 2(f)).

## 4. Discussion

Based on the fact that gene polymorphisms related to energetic expenditure and adipogenesis can be associated with obesogenic phenotypes in different populations, this study focused on the possible relationship between functional polymorphisms Gly482Ser in* PPARGC1A* and Pro12Ala in* PPARG2* with the accumulation and distribution of body fat and metabolic profile.

In the subjects of this study the mean in the BMI was of 26.8 kg/m^2^, which was similar to some populations [34–37] but greater than others [38–41]. In addition, we also noted in our cohort that BMI coexists with a fat mass percentage of 30.2 [37]. A recent report indicated that the distribution and quantity of adipose tissue and BMI are independent [42], moreover, in the present study we observed that accumulation and distribution of body fat tissue, levels of metabolic markers, soluble resistin and leptin, were higher when BMI increased, whereas levels of adiponectin and apolipoprotein A-1 decreased. Besides, we found increased metabolic markers when compared to other populations [34–38].

These findings support the previously stated hypothesis that a variation on BMI and the accumulation of body fat on individuals have a genetic factor involved. In addition, these data suggest that genetic factors might be involved in triggering altered metabolic profile in our population. This finding is consistent with Chandalia's proposal that even if two ethnic groups present the same adipose tissue proportion, histological and functional differences can be found as manifestations of diverse chronic comorbidities [42].

In this regard, different studies performed since the 80's consistently reported a relationship between the increase of adipose tissue and metabolic disorders [43]. In our study, we determined the correlation of metabolic markers levels with adiposity indexes, to evaluate the metabolic imbalance caused by adipose tissue. We found a positive correlation of glucose, insulin, HOMA-IR and lipid profile with adiposity indexes, whereas HDLc and apolipoprotein A-1 levels showed a negative correlation.

These data are consistent with a meta-analysis previously published by Fulop et al., who stated that through the increase in adipose tissue, the insulin resistance and cardiovascular risk also increased [44]. It is also consistent with diverse studies that have demonstrated the importance of adipose tissue as a metabolic regulator based upon its secreting capacity [45, 46] while changes in the amount and distribution of adipose tissue may favor the development of secondary chronic illness [44, 47].

With regard to susceptibility genes involved in adipose tissue differentiation and regulation, the polymorphism Pro12Ala in* PPARG2* was first described in 1997 by Chung-Jen et al. [48]. Further studies reported controversial results; nevertheless, most of them are consistent with the fact that allele 12Ala is associated with a greater risk of developing T2DM and gaining body weight on long term [49–51].

In our study, the allele 12Ala was observed to have a higher frequency when compared to populations from Palestine [52], Japan [19, 53], Turkey [54] and China [55]. This polymorphism was analyzed in different groups of Mexican people and the distribution found among them was similar to the present study [48, 56–59].

The supplementary analysis where we compare the carriers of different genotypes in* PPARG2* polymorphism, according to their classification by BMI, in groups with normal range and pre-obesity, showed no difference, except for HDLc levels in subjects with preobesity, while in the carriers of the phenotype Ala+ in obese group, an increase in metabolic markers was observed.

It is important to highlight that we are the first group reporting that 12Ala/Ala genotype carriers show increase in adiposity indexes, such as conicity and abdominal volume indexes and total adipose area, which are indicative of abdominal obesity, along with a difference in the distribution of Pro12Ala in* PPARG2* polymorphism among subjects with obesity when compared to nonobese subjects.

In this sense, such differences can be explained by the fact that* PPARG2* polymorphism increases its gene expression on abdominal adipose tissue, which in turn boost adipocyte differentiation and maturation in that body region and finally generates a metabolic pathogenic process [60–62]. The relevance of such finding lies in the existence of a potential pathogenic capacity of abdominal obesity that has led to consider it an important risk factor, both by itself and as a promoter of diseases that can generate comorbidity such as T2DM and cardiovascular disease.

On the other hand, abdominal adipose tissue is the main producer of the proinflammatory adipokines [63]. We found lower levels of total adiponectin, higher HOMA-IR and glucose in obese individuals carrying phenotype Ala+ than Ala− phenotype of* PPARG2 *gene. Such results have not been reported previously and more studies are required to look for a possible explanation. However, we hypothesize that it could be related to* PPARG2*'s transrepressing activity [64, 65].

The variations found in our results, cannot be exclusive for* PPARG2* and can be influenced by other protein interactions. Among the possible interacting molecules, PGC-1*α* stands out and has been proposed as an important orchestrator in glucose metabolism. The Gly482Ser* PPARGC1A* polymorphism has been associated with T2DM, hypertension, and Parkinson's disease [66–70].

Although the distribution of Gly482Ser in* PPARGC1A* showed no difference in our cohort, we found a heterozygous frequency of 39% and 38%, in normal range and preobese groups, respectively. This frequency is comparable with reports from other populations, while a 30% frequency was found for allele 482Ser, similar to others [66–68]. Previous studies report an association between allele 482Ser and the presence of T2DM in populations from China [71], Japan [72], Caucasians [73], and Korean [70] as well as a link with hypertension in German common people [11] and early insulin secretion in Pima Indians [12]. However, in our population there are no reports in this regard.

A relevant finding in our study was the presence of a greater magnitude in obesity indexes (e.g., body weight, BMI, body fat ratio, and total body fat mass) are present in carriers of genotype 482Ser/Ser. This could be explained by the fact that PGC-1*α* plays a role in adipocyte differentiation [74], which rises in subjects with adipose tissue hyperplasic and hypertrophic, individuals that can be catalogued as obese (i.e., BMI > 30 kg/m^2^).

Additionally, the involvement of* PPARGC1A* polymorphism in metabolic imbalance was evaluated. In this context, we found that allele 482Ser was associated with diminished glucose, triglycerides and apolipoprotein B (on heterozygote or homozygote polymorphic genotypes carriers) whilst, apolipoprotein A-1 levels was increased, at the same time, resistin and leptin levels were low on Gly− phenotype carriers. Nevertheless, no previous reports exist; hence, there is no referral for comparison. We suggest that carriers of allele 482Ser have a healthy metabolic profile.

In summary, we observed a heterogeneous profile in the polymorphic genotypes and phenotypes for both genes: 12Ala/Ala polymorphic genotype in* PPARG2,* an association with increasing magnitude of indicators of preferential accumulation of abdominal fat was found, whereas for polymorphic genotype 482Ser/Ser in* PPARGC1A* the connection is represented by a decreased in metabolic markers and adipokine levels. Unfortunately we were not able to show individuals carrying the polymorphic genotypes in both genes in this report. This implies we could not know the phenotype behavior.

These leads to the proposal of future investigations regarding these molecules since they could be taken into account as therapeutic targets and/or as surrogate markers of individuals that have an increased risk comorbidity development. This could lead to novel clinical and pharmacological approaches in glucose and adipose metabolism control.

## 5. Conclusions

In obese Mexican-Mestizos, the combination of alleles 482Ser in* PPARGC1A* and 12Pro in* PPARG2* presents a reduced metabolic risk profile; even adiposity indexes are increased.

## Figures and Tables

**Table 1:** Body dimensions, distribution of body fat storage, and metabolic markers profile according to BMI.

Classification	Normal range	Preobese	Obese
BMI (kg/m^2^)	[22.4 (18.5–24.9)]	[27.4 (25.1–29.6)]	[34.1 (30.3–45.4)]
*n*	153	144	78
Measurements			
Height (cm)^&^	164.7 ± 0.08	163.5 ± 0.09	163.4 ± 0.09
Body weight (kg)^*^	61.2 ± 8.6	73.5 ± 9.3	91.4 ± 13.9
Total body fat mass (kg)^*^	14.4 ± 5.7	22.9 ± 5.2	36.2 ± 8.9
Body fat ratio^*^	5.34 ± 2.18	8.56 ± 2.05	13.61 ± 3.38
Waist circumference (cm)^*^	78.3 ± 8.2	91.6 ± 8.1	106.5 ± 10.8
Hip circumference (cm)^*^	95.7 ± 6.1	102.6 ± 5.2	116.9 ± 10.2
WHR^+^	0.85 ± 0.12	0.86 ± 0.12	0.90 ± 0.10
WthR^*^	0.47 ± 0.04	0.56 ± 0.04	0.64 ± 0.06
Total adipose area (cm^2^)^*^	493 ± 105	672 ± 119	896 ± 183
Conicity index^*^	1.18 ± 0.10	1.25 ± 0.08	1.29 ± 0.09
Abdominal volume index (L)^*^	12.65 ± 2.54	17.04 ± 2.90	22.72 ± 4.55
Glucose (mg/dL)^*^	87 ± 11	96 ± 12	98 ± 13
Insulin (μIU/mL)^*^	14.1 ± 24.3	16.8 ± 13.9	25.1 ± 29.9
HOMA-IR^*^	2.97 ± 5.05	4.00 ± 3.41	5.89 ± 7.03
Triglycerides (mg/dL)^+^	115 ± 64	167 ± 103	153 ± 79
Total cholesterol (mg/dL)^+^	172 ± 37	192 ± 41	192 ± 31
HDLc (mg/dL)^&^	40.7 ± 16.0	37.2 ± 13.1	37.9 ± 14.2
LDLc (mg/dL)^*^	105 ± 35	113 ± 36	117 ± 26
VLDLc (mg/dL)^+^	23.1 ± 12.9	33.0 ± 20.5	30.4 ± 15.9
Apolipoprotein A-1 (mg/dL)^&^	118 ± 29	112 ± 22	117 ± 27
Apolipoprotein B (mg/dL)^+^	98 ± 28	124 ± 34	115 ± 27
Total sAdiponectin (ng/mL)^+^	7570 ± 340	3665 ± 293	4489 ± 351
sResistin (ng/mL)^+^	8.4 ± 2.5	9.7 ± 3.2	10.5 ± 3.7
sLeptin (ng/mL)^*^	17.6 ± 14.6	23.8 ± 15.4	57.5 ± 30.7
Abdominal obesity (%)	3.2	32.9	74.7
Dyslipidemia (%)	16.8	32.9	31.3

Data are means ± SD. One-way ANOVA test and Tukey post hoc test of normal range versus preobesity and obesity, and preobesity versus obesity, ^*^all measurements were different (*P* < 0.05), ^+^exception where only normal range versus obesity was different. ^&^No signifies differences were found. BMI: body mass index; WHR: waist-hip ratio; WthR: waist height ratio; HOMA-IR: homeostasis model assessment-insulin resistance; HDLc, LDLc, and VLDLc: high, low, and very low density lipoprotein cholesterol, respectively.

**Table 2:** Correlations of metabolic markers profile with body adiposity measurements.

Measurements	Glucose	Insulin	HOMA-IR	Triglycerides	Total cholesterol	HDLc	LDLc	VLDLc	Apo A-1	Apo B
	^ +^Correlation, *P*									
Body weight (kg)	**0.270** ^**^	**0.335** ^**^	**0.353** ^**^	**0.313** ^**^	**0.215** ^**^	−0.082	**0.116** ^*^	**0.308** ^**^	−0.113	**0.346** ^**^
	<0.0001	<0.0001	<0.0001	<0.0001	<0.0001	0.114	0.026	<0.0001	0.175	<0.0001

BMI (kg/m^2^)	**0.380** ^**^	**0.397** ^**^	**0.435** ^**^	**0.350** ^**^	**0.322** ^**^	−0.033	**0.195** ^**^	**0.344** ^**^	−0.018	**0.345** ^**^
	<0.0001	<0.0001	<0.0001	<0.0001	<0.0001	0.526	<0.0001	<0.0001	0.826	<0.0001

Total body fat mass (kg)	**0.339** ^**^	**0.324** ^**^	**0.366** ^**^	**0.284** ^**^	**0.284** ^**^	0.058	**0.173** ^**^	**0.284** ^**^	0.026	**0.317** ^**^
	<0.0001	<0.0001	<0.0001	<0.0001	<0.0001	0.322	0.003	<0.0001	0.834	0.006

Body fat ratio	**0.362** ^**^	**0.409** ^**^	**0.443** ^**^	**0.336** ^**^	**0.309** ^**^	−0.028	**0.191** ^**^	**0.330** ^**^	−0.021	**0.343** ^**^
	<0.0001	<0.0001	<0.0001	<0.0001	<0.0001	0.590	<0.0001	<0.0001	0.801	<0.0001

Waist circumference (cm)	**0.325** ^**^	**0.271** ^**^	**0.305** ^**^	**0.401** ^**^	**0.375** ^**^	**−0.124** ^*^	**0.268** ^**^	**0.396** ^**^	**−0.182** ^*^	**0.467** ^**^
	<0.0001	<0.0001	<0.0001	<0.0001	<0.0001	0.026	<0.0001	<0.0001	0.041	<0.0001

Hip circumference (cm)	**0.224** ^**^	**0.293** ^**^	**0.305** ^**^	**0.177** ^**^	**0.243** ^**^	0.042	**0.146** ^**^	**0.165** ^**^	0.011	**0.200** ^*^
	<0.0001	<0.0001	<0.0001	0.001	<0.0001	0.455	0.009	0.003	0.903	0.021

WHR	**0.325** ^**^	**0.271** ^**^	**0.305** ^**^	**0.401** ^**^	**0.375** ^**^	**−0.124** ^*^	**0.268** ^**^	**0.396** ^**^	**−0.182** ^*^	**0.467** ^**^
	<0.0001	<0.0001	<0.0001	<0.0001	<0.0001	0.026	<0.0001	<0.0001	0.041	<0.0001

Conicity index	**0.259** ^**^	**0.092**	**0.128** ^*^	**0.368** ^**^	**0.360** ^**^	**−0.156** ^**^	**0.281** ^**^	**0.366** ^**^	**−0.256** ^**^	**0.462** ^**^
	<0.0001	0.099	0.022	<0.0001	<0.0001	0.005	<0.0001	<0.0001	0.004	<0.0001

Total adipose area (cm^2^)	**0.325** ^**^	**0.271** ^**^	**0.305** ^**^	**0.401** ^**^	**0.375** ^**^	**−0.124** ^*^	**0.268** ^**^	**0.396** ^**^	**−0.182** ^*^	**0.467** ^**^
	<0.0001	<0.0001	<0.0001	<0.0001	<0.0001	0.026	<0.0001	<0.0001	0.041	<0.0001

Abdominal volume index (L)	**0.324** ^**^	**0.275** ^**^	**0.308** ^**^	**0.394** ^**^	**0.373** ^**^	**−0.117** ^*^	**0.266** ^**^	**0.390** ^**^	**−0.176** ^*^	**0.463** ^**^
	<0.0001	<0.0001	<0.0001	<0.0001	<0.0001	0.035	<0.0001	<0.0001	0.049	<0.0001

HOMA-IR: homeostasis model assessment-insulin resistance; Glucose, HDLc, LDLc, and VLDLc: high, low, and very low density lipoprotein cholesterol, respectively; Apo: apolipoprotein mg/dL. Insulin, μIU/mL. ^**^Significant correlation at 0.01 level. ^*^Significant correlation at 0.05 level. ^+^ *r* Pearson/Rho of Spearman correlations test.

**Table 3:** Distribution of Gly482Ser in *PPARGC1A* and Pro12Ala in *PPARG2* polymorphisms in Mexican-Mestizo population.

Study group	Genotype *n* (%)			Phenotype *n* (%)		Allele *n* (%)	
*PPARGC1A* Gly482Ser rs8192678	Gly/Gly (Ser−)	Gly/Ser	Ser/Ser (Gly−)	Gly+	Ser+	Gly	Ser

Normal range	77 (50)	60 (39)	16 (11)	137 (89)	76 (50)	214 (70)	92 (30)
Preobese	74 (51)	54 (38)	16 (11)	128 (89)	70 (49)	202 (70)	86 (30)
Obese	38 (49)	31 (40)	9 (11)	69 (89)	40 (51)	107 (70)	49 (30)
*P*	^ 1^NS			^ 2^NS	^ 2^NS	^ 5^NS	
Without obesity	151 (51)	114 (38)	32 (11)	265 (89)	146 (49)	416 (70)	178 (30)
*P*	^ 3^NS			^ 4^NS	^ 4^NS	^ 6^NS	

*PPARG2* Pro12Ala rs1801282	Pro/Pro (Ala−)	Pro/Ala	Ala/Ala (Pro−)	Pro+	Ala+	Pro	Ala

Normal range	117 (76)	34 (23)	2 (1)	151 (99)	36 (24)	268 (87)	38 (13)
Preobese	117 (81)	27 (19)	0 (0)	144 (100)	27 (19)	261 (90)	27 (10)
Obese	61 (78)	13 (17)	4 (5)	74 (95)	17 (22)	135 (87)	21 (13)
*P*	^ 1^ **0.047**			^ 2^ **0.014**	^ 2^NS	^ 5^NS	
Without obesity	234 (78)	61 (21)	2 (1)	295 (99)	63 (22)	529 (89)	65 (11)
*P*	^ 3^ **0.017**			^ 4^ **0.019**	^ 4^NS	^ 6^NS	

*n* = 375; NS: not significant; categorical variables were analyzed using χ^2^ or Fisher exact test, accordingly. Comparisons: ^1^normal range (BMI 18.5–24.9 kg/m^2^), preobese (BMI 25–29.9 kg/m^2^), and obese (BMI ≥ 30 kg/m^2^) by genotype; ^2^normal range, preobese, and obese by phenotype; ^3^obese and without obesity (BMI: 18.5–29.9 kg/m^2^) by genotype; ^4^obese and without obesity by phenotype; ^5^normal range, preobese, and obese by allele; ^6^normal range and without obesity by allele.

**Table:** (a) Normal range BMI subjects

Measurements	Polymorphism *PPARGC1A *				Polymorphism *PPARG2 *			
	Genotypes			Phenotype	Genotypes			Phenotype
	Gly/Gly	Gly/Ser	Ser/Ser	Ser+	Pro/Pro	Pro/Ala	Ala/Ala	Ala+
*n*	77	60	16	76	117	34	2	36
Height (cm)	164.6 ± 8.3	164.7 ± 9.3	165.5 ± 10.5	164.9 ± 9.5	163.9 ± 9.0	167.5 ± 7.8	165.0 ± 11.3	167.3 ± 7.8
Body weight (kg)	61.0 ± 7.7	60.9 ± 9.5	62.9 ± 9.2	61.4 ± 9.4	60.7 ± 8.9	62.9 ± 6.8	59.5 ± 17.2	62.7 ± 7.2
BMI (kg/m^2^)	22.49 ± 1.7	22.32 ± 1.6	22.77 ± 1.6	22.4 ± 1.6	22.48 ± 1.6	22.40 ± 1.6	21.55 ± 3.3	22.3 ± 1.7
Total body fat mass (kg)	14.1 ± 5.2	15.0 ± 6.5	13.8 ± 5.8	14.7 ± 6.3	14.6 ± 5.8	13.9 ± 5.3	15.6 ± 11.7	14.0 ± 5.6
Body fat ratio	5.23 ± 1.93	5.60 ± 2.48	4.88 ± 2.25	5.46 ± 2.43	5.46 ± 2.21	4.95 ± 2.08	5.48 ± 3.56	4.98 ± 2.11
Waist circumference (cm)	78.1 ± 8.1	78.1 ± 8.4	80.1 ± 7.9	78.5 ± 8.2	77.7 ± 8.1	80.4 ± 7.9	70.4 ± 6.8	80.1 ± 8.0
Hip circumference (cm)	95.5 ± 4.8	95.9 ± 7.8	95.3 ± 4.5	95.8 ± 7.1	95.8 ± 6.5	95.4 ± 4.5	87.7 ± 5.3	95.2 ± 4.6
WHR	0.82 ± 0.08	0.80 ± 0.14	0.84 ± 0.07	0.81 ± 0.13	0.80 ± 0.11	0.84 ± 0.08	0.79 ± 0.02	0.84 ± 0.09
WthR	0.47 ± 0.04	0.47 ± 0.05	0.48 ± 0.04	0.48 ± 0.05	0.48 ± 0.05	0.48 ± 0.05	0.45 ± 0.05	0.48 ± 0.05
Total adipose area (cm^2^)	490 ± 104	490 ± 111	515 ± 97	496 ± 108	486 ± 105	519 ± 104	394 ± 92	515 ± 105
Conicity index	1.17 ± 0.09	1.18 ± 0.12	1.19 ± 0.08	1.19 ± 0.12	1.18 ± 0.11	1.20 ± 0.11	1.18 ± 0.01	1.20 ± 0.11
Abdominal volume index (L)	12.5 ± 2.49	12.6 ± 2.71	13.1 ± 2.33	12.7 ± 2.62	12.5 ± 2.5	13.3 ± 2.5	10.1 ± 0.02	13.1 ± 2.54
**Glucose (mg/dL)** ^*^	87 ± 9.3	**89 ± 12.1**	**80 ± 13.4**	87 ± 12.7	87 ± 11.8	85 ± 8.6	79 ± 4.24	85 ± 8.5
Insulin (μIU/mL)	11.3 ± 12.9	18.4 ± 35.5	10.9 ± 7.5	16.9 ± 31.8	14.9 ± 27.3	11.4 ± 9.4	10.2 ± 4.1	11.3 ± 9.2
HOMA-IR	2.40 ± 2.89	3.95 ± 7.22	1.89 ± 1.11	3.54 ± 6.51	3.51 ± 5.67	2.40 ± 2.00	1.98 ± 0.70	2.20 ± 1.90
Triglycerides (mg/dL)	117 ± 70	113 ± 57	114 ± 66	113 ± 58	115 ± 68	118 ± 53	74 ± 6	115 ± 53
Total cholesterol (mg/dL)	172 ± 32	173 ± 43	171 ± 39	172 ± 42	172 ± 36	173 ± 42	159 ± 60	172 ± 42
HDLc (mg/dL)	39 ± 17.6	41 ± 16.1	41 ± 17.3	41 ± 16.2	41 ± 17.0	38 ± 16.9	34 ± 5.7	16 ± 2.8
LDLc (mg/dL)	106 ± 29.4	105 ± 41.6	105 ± 42.2	105 ± 41.5	104 ± 35.3	107 ± 37.7	110 ± 53.9	37 ± 6.3
VLDLc (mg/dL)	23.5 ± 14.1	22.7 ± 11.4	22.9 ± 13.4	22.7 ± 11.8	23.1 ± 13.6	23.6 ± 10.7	14.9 ± 1.3	23.2 ± 10.6
Apolipoprotein A-1 (mg/dL)	118 ± 24.4	116 ± 35.9	125 ± 20.3	118 ± 24.4	117 ± 30.9	123 ± 21.7	112 ± 11.7	123 ± 21.7
Apolipoprotein B (mg/dL)	102 ± 30.1	97 ± 28.9	89 ± 20.9	102 ± 30.1	99 ± 29.0	96 ± 27.4	98 ± 12.8	97 ± 10.3
Total sAdiponectin (ng/mL)	8119 ± 4358	6540 ± 1808	10361 ± 4378	8119 ± 4358	7756 ± 3073	7130 ± 5673	5046 ± 2326	5001 ± 2236
sResistin (ng/mL)^&^	8.1 ± 2.5	8.4 ± 2.3	9.7 ± 3.8	8.1 ± 2.5	7.9 ± 2.2	11.2 ± 1.5	10.7 ± 1.9	11.3 ± 1.5
sLeptin (ng/mL)	18.5 ± 14.9	18.0 ± 15.1	14.6 ± 3.2	18.4 ± 14.9	16.2 ± 14.5	20.8 ± 17.4	33.2 ± 11.9	23.9 ± 15.5

*n* = 153. Normal range BMI 18.5–24.9 kg/m^2^. Data are means ± SD. ^*^Gly/Ser versus Ser/Ser genotypes, *P* = 0.022.  ^&^ALA− versus ALA+ phenotypes *P* = 0.009.

BMI: body mass index; WHR: waist-hip ratio; WthR: waist height ratio; HOMA-IR: Homeostasis model assessment-insulin resistance; HDLc, LDLc, and VLDLc: high, low, and very low density lipoprotein cholesterol, respectively.

**Table:** (b) Preobese range BMI subjects

Measurements	Polymorphism *PPARGC1A *				Polymorphism *PPARG2 *			
	Genotypes			Phenotype	Genotypes			Phenotype
	Gly/Gly	Gly/Ser	Ser/Ser	Ser+	Pro/Pro	Pro/Ala	Ala/Ala	Ala+
*n*	74	54	16	70	117	27	0	27
Height (cm)	163.8 ± 8.8	164.8 ± 9.77	157.9 ± 6.6	163.2 ± 9.6	164.2 ± 8.9	160.5 ± 9.8	—	160.5 ± 9.8
Body weight (kg)	74.0 ± 9.3	74.2 ± 9.7	68.5 ± 5.7	72.8 ± 9.3	74.0 ± 8.9	71.3 ± 10.6	—	71.3 ± 10.6
BMI (kg/m^2^)	27.50 ± 1.50	27.20 ± 1.32	27.48 ± 1.43	27.27 ± 1.34	27.35 ± 1.42	27.55 ± 1.45	—	27.55 ± 1.45
Total body fat mass (kg)	23.8 ± 5.1	22.4 ± 5.5	21.4 ± 4.3	22.1 ± 5.2	22.6 ± 5.4	23.8 ± 4.4	—	23.8 ± 4.4
Body fat ratio	8.78 ± 2.04	8.28 ± 2.17	8.61 ± 1.78	8.37 ± 2.06	8.40 ± 2.16	9.14 ± 1.51	—	9.14 ± 1.51
Waist circumference (cm)	92.3 ± 8.4	91.2 ± 8.2	90.1 ± 6.7	91.0 ± 7.8	91.6 ± 7.7	91.5 ± 9.8	—	91.5 ± 9.8
Hip circumference (cm)	103.6 ± 5.1	101.9 ± 5.5	100.8 ± 3.8	101.6 ± 5.1	102.6 ± 5.3	102.8 ± 4.5	—	102.8 ± 4.5
WHR	0.90 ± 0.14	0.90 ± 0.08	0.90 ± 0.07	0.89 ± 0.08	0.89 ± 0.12	0.89 ± 0.08	—	0.89 ± 0.08
WthR	0.56 ± 0.04	0.55 ± 0.04	0.57 ± 0.03	0.56 ± 0.04	0.56 ± 0.04	0.58 ± 0.04	—	0.58 ± 0.04
Total adipose area (cm^2^)	682 ± 125	667 ± 18	649 ± 95	662 ± 112	672 ± 112	673 ± 149	—	673 ± 149
Conicity index	1.26 ± 0.08	1.25 ± 0.09	1.26 ± 0.07	1.25 ± 0.09	1.25 ± 0.08	1.27 ± 0.09	—	1.27 ± 0.09
Abdominal volume index (L)	17.30 ± 3.08	16.90 ± 2.87	16.43 ± 2.29	16.78 ± 2.73	17.04 ± 2.74	17.10 ± 3.7	—	17.07 ± 3.70
Glucose (mg/dL)	97 ± 12.1	95 ± 11.7	92 ± 13.5	94 ± 12.1	95 ± 11.3	97 ± 15.4	—	97 ± 15.4
Insulin (μIU/mL)	15.9 ± 9.9	18.9 ± 19.1	13.3 ± 5.9	17.6 ± 17.1	17.3 ± 14.9	14.5 ± 7.5	—	14.5 ± 7.5
HOMA-IR	3.91 ± 2.54	4.43 ± 4.63	3.06 ± 1.48	4.11 ± 4.15	4.12 ± 3.67	3.52 ± 1.99	—	3.53 ± 1.99
**Triglycerides (mg/dL)** ^*^	**186 ± 124**	153 ± 74	133 ± 60	**148 ± 71**	173 ± 108	145 ± 76	—	145 ± 76
Total cholesterol (mg/dL)	195 ± 43	189 ± 40	188 ± 31	189 ± 38	193 ± 44	187 ± 25	—	187 ± 25
**HDLc (mg/dL)** ^ &^	37 ± 13.6	36 ± 13.0	32 ± 12.2	36 ± 12.7	**36 ± 12.7**	41 ± 14.1	—	**41 ± 14.1**
LDLc (mg/dL)	111 ± 32.5	115 ± 41.3	115 ± 35.4	115 ± 39.7	114 ± 38.1	107 ± 25.6	—	107 ± 25.6
VLDLc (mg/dL)	36.2 ± 24.8	30.5 ± 14.7	26.8 ± 12.1	29.6 ± 14.2	34.0 ± 21.6	29.1 ± 15.3	—	29.1 ± 15.3
Apolipoprotein A-1 (mg/dL)	109 ± 15.6	120 ± 31.5	108 ± 6.7	118 ± 29.1	110 ± 15.8	124 ± 37.5	—	124 ± 37.5
Apolipoprotein B (mg/dL)	129 ± 40.5	117 ± 23.0	113 ± 9.0	117 ± 21.4	125 ± 35.4	121 ± 30.9	—	121 ± 30.9
Total sAdiponectin (ng/mL)	3929 ± 3145	3563 ± 2715	3283 ± 3107	3485 ± 2801	3646 ± 2745	3721 ± 3516	—	3721 ± 3516
sResistin (ng/mL)	8.9 ± 2.6	10.6 ± 3.8	10.5 ± 4.9	10.6 ± 3.8	9.3 ± 3.4	10.6 ± 2.8	—	10.6 ± 2.8
sLeptin (ng/mL)	24.1 ± 14.3	20.2 ± 14.9	31.3 ± 16.7	23.6 ± 16.1	23.2 ± 16.0	26.0 ± 13.5	—	26.0 ± 13.5

*n* = 144. Preobese range, BMI 25.0–29.9 kg/m^2^. Data are means ± SD. ^*^Ser− versus Ser+ phenotypes, P = 0.030. ^&^Ala− versus Ala+ phenotypes,   *P* = 0.039.

**Table:** (c) Obese range BMI subjects

Measurements	Polymorphism *PPARGC1A *				Polymorphism *PPARG2 *			
	Genotypes			Phenotype	Genotypes			Phenotype
	Gly/Gly	Gly/Ser	Ser/Ser	Ser+	Pro/Pro	Pro/Ala	Ala/Ala	Ala+
*n*	38	31	9	40	61	13	4	17
Height (cm)	162.9 ± 9.3	162.1 ± 9.8	170.5 ± 1.3	164.0 ± 10.6	164.2 ± 9.9	159.2 ± 8.8	166.8 ± 13.4	160.9 ± 10.1
**Body weight (kg)** ^*^	**90.6 ± 11.8**	**87.4 ± 13.4**	**108.9 ± 10.8**	92.2 ± 15.6	91.9 ± 14.6	89.7 ± 10.9	89.7 ± 12.9	89.7 ± 11.0
**BMI (kg/** **m** ^2^ **)** ^*^	**34.08 ± 3.34**	**33.18 ± 0.55**	**37.80 ± 5.30**	34.21 ± 4.38	34.02 ± 4.03	35.41 ± 3.47	32.18 ± 1.49	34.64 ± 3.38
**Total body fat mass (kg)** ^*^	**34.4 ± 7.8**	**34.3 ± 7.1**	**47.7 ± 9.2**	37.9 ± 9.7	36.7 ± 8.8	35.4 ± 10.9	32.4 ± 4.5	34.5 ± 9.4
**Body fat ratio** ^*^	**13.12 ± 3.23**	**13.19 ± 2.76**	**16.48 ± 0.42**	14.06 ± 3.52	13.73 ± 3.32	13.87 ± 4.04	11.81 ± 2.35	13.2 ± 3.7
Waist circumference (cm)	106.4 ± 11.7	103.2 ± 9.6	110.9 ± 9.6	104.9 ± 9.9	105.7 ± 10.9	102.2 ± 10.5	113.1 ± 7.9	105.3 ± 10.8
**Hip circumference (cm)** ^*^	**115.8 ± 9.6**	**115.6 ± 8.2**	**127.6 ± 14.3**	118.1 ± 10.7	117.5 ± 10.3	116.7 ± 0.4	110.0 ± 5.9	114.8 ± 9.6
**WHR** ^°&^	**0.94 ± 0.12**	0.89 ± 0.08	**0.77 ± 0.31**	**0.87 ± 0.16**	0.89 ± 0.15	0.90 ± 0.11	1.03 ± 0.10	0.93 ± 0.12
WthR	0.65 ± 0.07	0.63 ± 0.07	0.65 ± 0.08	0.63 ± 0.07	0.64 ± 0.07	0.65 ± 0.07	0.68 ± 0.04	0.65 ± 0.07
Total adipose area (cm^2^)	911 ± 198	855 ± 161	984 ± 167	882 ± 168	898 ± 186	838 ± 167	1021 ± 138	891 ± 176
**Conicity index** ^ #^	1.31 ± 0.11	1.28 ± 0.09	1.26 ± 0.07	1.28 ± 0.08	**1.29 ± 0.08**	**1.25 ± 0.14**	**1.42 ± 0.05**	1.30 ± 0.15
Abdominal volume index (L)	23.08 ± 4.92	21.66 ± 3.97	24.98 ± 4.25	22.4 ± 4.2	22.8 ± 4.6	21.3 ± 4.1	25.7 ± 3.5	22.6 ± 4.4
**Glucose (mg/dL)** ^ +^	98 ± 15.2	97 ± 11.4	92 ± 11.2	96 ± 11.4	**96 ± 13.3**	104 ± 13.3	96 ± 9.0	**103 ± 13.8**
**Insulin (μIU/mL)** ^ +^	21.4 ± 22.0	27.2 ± 36.8	33.8 ± 33.3	28.7 ± 35.7	**22.6 ± 20.4**	40.8 ± 57.3	11.9 ± 7.0	**39.5 ± 55.2**
**HOMA-IR** ^ +∆^	5.45 ± 5.79	6.59 ± 9.00	5.35 ± 3.21	6.34 ± 8.13	**5.13 ± 4.27**	**10.38 ± 13.9**	2.92 ± 1.96	**10.05 ± 13.4**
**Triglycerides (mg/dL)** ^ &^	**171 ± 103**	130 ± 40	151 ± 42	**135 ± 41**	155 ± 85	142 ± 52	149 ± 58	144 ± 52
Total cholesterol (mg/dL)	194 ± 26	194 ± 38	178 ± 23	191 ± 36	191 ± 33	193 ± 24	219 ± 21	199 ± 25
HDLc (mg/dL)	36 ± 10.3	38 ± 16.9	43 ± 18.8	39 ± 17.2	36 ± 14.8	40 ± 10.9	48 ± 13.4	42 ± 11.6
**LDLc (mg/dL)** ^∞^	120 ± 27.4	118 ± 25.1	99 ± 26.4	114 ± 26.3	**114 ± 26.7**	121 ± 21.4	**150 ± 27.7**	128 ± 25.4
VLDLc (mg/dL)	33.9 ± 20.7	26.2 ± 8.2	30.3 ± 8.4	27.2 ± 8.3	30.9 ± 17.2	28.5 ± 10.5	30.0 ± 11.8	28.8 ± 10.5
**Apolipoprotein A1 (mg/dL)** ^°&^	**106 ± 16.9**	**123 ± 28.7**	**148 ± 37.9**	128 ± 31.5	116 ± 29.3	124 ± 12.4	112 ± 17.9	119 ± 15.0
**Apolipoprotein B (mg/dL)** ^*^	**121 ± 25.8**	**115 ± 16.8**	**80 ± 46.8**	108 ± 28.3	116 ± 20.9	87 ± 54.7	134 ± 37.5	107 ± 50.8
**Total sAdiponectin (ng/mL)** ^ +^	4421 ± 3452	3897 ± 1769	5946 ± 5929	4553 ± 3646	**4598 ± 3779**	3596 ± 2364	4861 ± 1613	**3210 ± 1466**
sResistin (ng/mL)	10.3 ± 4.1	9.3 ± 2.0	14.3 ± 2.3	10.8 ± 3.1	10.5 ± 3.3	8.0 ± 3.7	11.7 ± 6.7	10.8 ± 5.7
sLeptin (ng/mL)	55.8 ± 31.1	54.8 ± 26.4	72.6 ± 41.1	58.9 ± 30.7	55.4 ± 30.9	69.5 ± 30.7	57.4 ± 26.7	66.1 ± 29.1

*n* = 78. Obese range, BMI >30.0 kg/m^2^. Data are means ± SD. ^*^Gly/Gly versus Gly/Ser and Ser/Ser genotypes, °Gly/Gly versus Ser/Ser genotypes, ^&^Ser− versus Ser+ phenotypes, ^#^Pro/Pro versus Pro/Ala and Ala/Ala genotypes, ^∆^Pro/Pro versus Pro/Ala genotypes, ^∞^Pro/Pro versus Ala/Ala genotypes, ^+^Ala− versus Ala+ phenotypes, P < 0.05.

## References

[1] Martyn J. A. J., Kaneki M., Yasuhara S. (2008). Obesity-induced insulin resistance and hyperglycemia: etiologic factors and molecular mechanisms. *Anesthesiology*.

[2] van Greevenbroek M. M. J., Schalkwijk C. G., Stehouwer C. D. A. (2013). Obesity-associated low-grade inflammation in type 2 diabetes mellitus: causes and consequences. *Netherlands Journal of Medicine*.

[3] Xia Q., Grant S. F. A. (2013). The genetics of human obesity. *Annals of the New York Academy of Sciences*.

[4] Chung W. K., Leibel R. L. (2008). Considerations regarding the genetics of obesity. *Obesity*.

[5] Naukkarinen J., Rissanen A., Kaprio J., Pietiläinen K. H. (2012). Causes and consequences of obesity: the contribution of recent twin studies. *International Journal of Obesity*.

[6] Dahlman I., Arner P. (2010). Genetics of adipose tissue biology. *Progress in Molecular Biology and Translational Science*.

[7] Esterbauer H., Oberkofler H., Krempler F., Patsch W. (1999). Human peroxisome proliferator activated receptor gamma coactivator 1 (*PPARGC1*) gene: cDNA sequence, genomic organization, chromosomal localization, and tissue expression. *Genomics*.

[8] Ek J., Andersen G., Urhammer S. A. (2001). Mutation analysis of peroxisome proliferator-activated receptor-gamma coactivator-1 (PGC-1) and relationships of identified amino acid polymorphisms to Type II diabetes mellitus. *Diabetologia*.

[9] Zhang S.-L., Lu W.-S., Yan L. (2007). Association between peroxisome proliferator-activated receptor-*γ* coactivator-1*α* gene polymorphisms and type 2 diabetes in southern Chinese population: role of altered interaction with myocyte enhancer factor 2C. *Chinese Medical Journal*.

[10] Sainani G. S., Karatela R. A. (2009). Plasma leptin in insulin-resistant and insulin-nonresistant coronary artery disease and its association with cardio-metabolic risk factors among Asian Indians. *Metabolic Syndrome and Related Disorders*.

[11] Oberkofler H., Hölzl B., Esterbauer H. (2003). Peroxisome proliferator-activated receptor-*γ* coactivator-1 gene locus: associations with hypertension in middle-aged men. *Hypertension*.

[12] Muller Y. L., Bogardus C., Pedersen O., Baier L. (2003). A Gly482Ser missense mutation in the peroxisome proliferator-activated receptor *γ* coactivator-1 is associated with altered lipid oxidation and early insulin secretion in Pima Indians. *Diabetes*.

[13] Francès F., Verdú F., Portolés O. (2008). *PPAR-α* L162V and *PGC-1* G482S gene polymorphisms, but not *PPAR-γ* P12A, are associated with alcohol consumption in a Spanish Mediterranean population. *Clinica Chimica Acta*.

[14] Lefterova M. I., Haakonsson A. K., Lazar M. A., Mandrup S. (2014). PPAR*γ* and the global map of adipogenesis and beyond. *Trends in Endocrinology & Metabolism*.

[15] Ahmadian M., Suh J. M., Hah N. (2013). PPARgamma signaling and metabolism: the good, the bad and the future. *Nature Medicine*.

[16] Floyd Z. E., Stephens J. M. (2012). Controlling a master switch of adipocyte development and insulin sensitivity: covalent modifications of PPAR*γ*. *Biochimica et Biophysica Acta: Molecular Basis of Disease*.

[17] Cinti S. (2009). Transdifferentiation properties of adipocytes in the adipose organ. *American Journal of Physiology: Endocrinology and Metabolism*.

[18] Yamamoto J., Kageyama S., Nemoto M. (2002). PPAR*γ*2 Pro12Ala polymorphism and insulin resistance in Japanese hypertensive patients. *Hypertension Research*.

[19] Wang F., Tahara T., Arisawa T. (2008). Polymorphism of peroxisome proliferator-activated receptor gamma is not associated to Japanese ulcerative colitis. *Hepato-Gastroenterology*.

[20] Stumvoll M., Häring H. (2002). The peroxisome proliferator-activated receptor-*γ*2 Pro12Ala polymorphism. *Diabetes*.

[21] Gorodezky C., Alaez C., Vázquez-García M. N. (2001). The genetic structure of Mexican Mestizos of different locations: tracking back their origins through MHC genes, blood group systems, and microsatellites. *Human Immunology*.

[22] World-Health-Organization (2012). *Obesity: Preventing and Managing the Global Epidemic*.

[23] Lukaski H. C. (2001). Body mass index, bioelectrical impedance, and body composition. *Nutrition*.

[24] Lukaski H. C. (2003). Regional bioelectrical impedance analysis: applications in health and medicine. *Acta Diabetologica*.

[25] Ness-Abramof R., Apovian C. M. (2008). Waist circumference measurement in clinical practice. *Nutrition in Clinical Practice*.

[26] Miller D. N., Bryant J. E., Madsen E. L., Ghiorse W. C. (1999). Evaluation and optimization of DNA extraction and purification procedures for soil and sediment samples. *Applied and Environmental Microbiology*.

[27] Guzman-Ornelas M.-O., Chavarria-Avila E., Munoz-Valle J.-F. (2012). Association of ADIPOQ +45T>G polymorphism with body fat mass and blood levels of soluble adiponectin and inflammation markers in a Mexican-Mestizo population. *Diabetes, Metabolic Syndrome and Obesity: Targets and Therapy*.

[28] Guerrero-Romero F., Rodríguez-Morán M. (2003). Abdominal volume index. An anthropometry-based index for estimation of obesity is strongly related to impaired glucose tolerance and type 2 diabetes mellitus. *Archives of Medical Research*.

[29] Garaulet M., Hernández-Morante J. J., Tébar F. J., Zamora S., Canteras M. (2006). Two-dimensional predictive equation to classify visceral obesity in clinical practice. *Obesity*.

[30] Mamtani M. R., Kulkarni H. R. (2005). Predictive performance of anthropometric indexes of central obesity for the risk of type 2 diabetes. *Archives of Medical Research*.

[31] Matthews D. R., Hosker J. P., Rudenski A. S., Naylor B. A., Treacher D. F., Turner R. C. (1985). Homeostasis model assessment: insulin resistance and *β*-cell function from fasting plasma glucose and insulin concentrations in man. *Diabetologia*.

[32] Friedewald W. T., Levy R. I., Fredrickson D. S. (1972). Estimation of the concentration of low-density lipoprotein cholesterol in plasma, without use of the preparative ultracentrifuge.. *Clinical Chemistry*.

[33] Hardy G. H. (1908). Mendelian proportions in a mixed population. *Science*.

[34] de Oliveira E. P., de Lima M. D. D. A., de Souza M. L. A. (2007). Metabolic syndrome, its phenotypes, and insulin resistance by HOMA-IR. *Arquivos Brasileiros de Endocrinologia e Metabologia*.

[35] Benozzi S., Ordonez F., Polini N., Alvarez C., Sellest J., Coniglio R. I. (2009). Insulin-resistance and metabolic syndrome in patients with coronary heart disease defined by angiography. *Medicina*.

[36] Dalan R., Jong M., Chan S. P. (2010). High-sensitivity C-reactive protein concentrations among patients with and without diabetes in a multiethnic population of Singapore: CREDENCE Study. *Diabetes, Metabolic Syndrome and Obesity: Targets and Therapy*.

[37] Chu S. H., Lee M. K., Ahn K. Y. (2012). Chemerin and adiponectin contribute reciprocally to metabolic syndrome. *PLoS ONE*.

[38] Tsuriya D., Morita H., Morioka T. (2011). Significant correlation between visceral adiposity and high-sensitivity C-reactive protein (hs-CRP) in Japanese subjects. *Internal Medicine*.

[39] Ou W., Liu X., Shen Y. (2014). Association of CVD candidate gene polymorphisms with ischemic stroke and cerebral hemorrhage in chinese individuals. *PLoS ONE*.

[40] Vasudevan D., Stotts A. L., Mandayam S., Omegie L. A. (2010). Comparison of BMI and anthropometric measures among South Asian Indians using standard and modified criteria. *Public Health Nutrition*.

[41] Harbron J., van der Merwe L., Zaahl M. G., Kotze M. J., Senekal M. (2014). Fat mass and obesity-associated (FTO) gene polymorphisms are associated with physical activity, food intake, eating behaviors, psychological health, and modeled change in body mass index in overweight/obese Caucasian adults. *Nutrients*.

[42] Chandalla M., Lin P., Seenivasan T. (2007). Insulin resistance and body fat distribution in South Asian men compared to Caucasian men. *PLoS ONE*.

[43] Kaplan N. M. (1989). The deadly quartet. Upper-body obesity, glucose intolerance, hypertriglyceridemia, and hypertension. *Archives of Internal Medicine*.

[44] Fulop T., Tessier D., Carpentier A. (2006). The metabolic syndrome. *Pathologie Biologie*.

[45] Karastergiou K., Mohamed-Ali V. (2010). The autocrine and paracrine roles of adipokines. *Molecular and Cellular Endocrinology*.

[46] Galic S., Oakhill J. S., Steinberg G. R. (2010). Adipose tissue as an endocrine organ. *Molecular and Cellular Endocrinology*.

[47] de Luca C., Olefsky J. M. (2008). Inflammation and insulin resistance. *FEBS Letters*.

[48] Chung-Jen Y., Beamer B. A., Negri C. (1997). Molecular scanning of the human peroxisome proliferator activated receptor *γ* (hPPAR*γ*) gene in diabetic Caucasians: identification of a Pro12Ala PPAR*γ*2 missense mutation. *Biochemical and Biophysical Research Communications*.

[49] Deeb S. S., Fajas L., Nemoto M. (1998). A Pro12Ala substitution in PPAR*γ*2 associated with decreased receptor activity, lower body mass index and improved insulin sensitivity. *Nature Genetics*.

[50] Kuliczkowska J., Filus A., Trzmiel A. (2008). PPAR-*γ*2 Pro12Ala polymorphism in the population of obese and non-obese men of the city of Wroclaw. *Endokrynologia Polska*.

[51] West N. A., Haan M. N., Morgenstern H. (2010). The PPAR-gamma Pro12Ala polymorphism and risk of cognitive impairment in a longitudinal study. *Neurobiology of Aging*.

[52] Ereqat S., Nasereddin A., Azmi K., Abdeen Z., Amin R. (2009). Impact of the pro12Ala polymorphism of the PPAR-gamma 2 gene on metabolic and clinical characteristics in the palestinian type 2 diabetic patients. *PPAR Research*.

[53] Aoyagi Y., Nagata S., Kudo T. (2010). Peroxisome proliferator-activated receptor *γ* 2 mutation may cause a subset of ulcerative colitis. *Pediatrics International*.

[54] Atug O., Tahan V., Eren F. (2008). Pro12Ala polymorphism in the peroxisome proliferator-activated receptor-gamma (PPAR*γ*) gene in inflammatory bowel disease. *Journal of Gastrointestinal and Liver Diseases*.

[55] Shrestha U. K., Karimi O., Crusius J. B. A. (2010). Distribution of peroxisome proliferator-activated receptor-gamma polymorphisms in Chinese and Dutch patients with inflammatory bowel disease. *Inflammatory Bowel Diseases*.

[56] Duran-Gonzalez J., Ortiz I., Gonzales E. (2011). Association study of candidate gene polymorphisms and obesity in a young Mexican-American population from South Texas. *Archives of Medical Research*.

[57] Estrada-Velasco B. I., Cruz M., Madrid-Marina V., Martínez-Nava G. A., Gomez-Zamudio J., Burguete-García A. I. (2013). *IRS1*, *TCF7L2*, *ADRB1*, *PPARG*, and *HHEX* polymorphisms associated with atherogenic risk in Mexican population. *BioMed Research International*.

[58] Gamboa-Meléndez M. A., Huerta-Chagoya A., Moreno-Macías H. (2012). Contribution of common genetic variation to the risk of type 2 diabetes in the Mexican Mestizo population. *Diabetes*.

[59] Martínez-Gómez L. E., Cruz M., Martínez-Nava G. A. (2011). A replication study of the IRS1, CAPN10, TCF7L2, and PPARG gene polymorphisms associated with type 2 diabetes in two different populations of Mexico. *Annals of Human Genetics*.

[60] Berger J., Moller D. E. (2002). The mechanisms of action of PPARs. *Annual Review of Medicine*.

[61] Berger J. P., Akiyama T. E., Meinke P. T. (2005). ARs-ARtherapeutic targets for metabolic disease. *Trends in Pharmacological Sciences*.

[62] Fève B. (2005). Adipogenesis: cellular and molecular aspects. *Best Practice & Research Clinical Endocrinology & Metabolism*.

[63] Guerro-Millo M. (2004). Adipose tissue and adipokines: for better or worse. *Diabetes & Metabolism*.

[64] Mansour M. (2014). The roles of peroxisome proliferator-activated receptors in the metabolic syndrome. *Progress in Molecular Biology and Translational Science*.

[65] Monsalve F. A., Pyarasani R. D., Delgado-Lopez F., Moore-Carrasco R. (2013). Peroxisome proliferator-activated receptor targets for the treatment of metabolic diseases. *Mediators of Inflammation*.

[66] Bhat A., Koul A., Rai E., Sharma S., Dhar M. K. (2007). PGC-1*α* Thr394Thr and Gly482Ser variants are significantly associated with T2DM in two North Indian populations: a replicate case-control study. *Human Genetics*.

[67] Clark J., Reddy S., Zheng K., Betensky R. A., Simon D. K. (2011). Association of PGC-1alpha polymorphisms with age of onset and risk of Parkinson's disease. *BMC Medical Genetics*.

[68] Ginevičiene V., Pranculis V., Jakaitiene V., Milašius V., Kučinskas V. (2011). Genetic variation of the human ACE and ACTN3 genes and their association with functional muscle properties in Lithuanian elite athletes. *Medicina*.

[69] Ingelsson E., Bennet L., Ridderstråle M., Söderström M., Råstam L., Lindblad U. (2008). The *PPARGC1A* Gly482Ser polymorphism is associated with left ventricular diastolic dysfunction in men. *BMC Cardiovascular Disorders*.

[70] Kim J. H., Shin H. D., Park B. L. (2005). Peroxisome proliferator-activated receptor gamma coactivator 1 alpha promoter polymorphisms are associated with early-onset type 2 diabetes mellitus in the Korean population. *Diabetologia*.

[71] Hsieh M.-C., Lin K.-D., Tien K.-J. (2010). Common polymorphisms of the peroxisome proliferator-activated receptor-*γ* (Pro12Ala) and peroxisome proliferator-activated receptor-*γ* coactivator-1 (Gly482Ser) and the response to pioglitazone in Chinese patients with type 2 diabetes mellitus. *Metabolism: Clinical and Experimental*.

[72] Okauchi Y., Iwahashi H., Okita K. (2008). PGC-1*α* Gly482Ser polymorphism is associated with the plasma adiponectin level in type 2 diabetic men. *Endocrine Journal*.

[73] Andrulionytè L., Zacharova J., Chiasson J.-L., Laakso M. (2004). Common polymorphisms of the *PPAR-γ*2 (*Pro12Ala*) and *PGC-*1*α* (*Gly482Ser*) genes are associated with the conversion from impaired glucose tolerance to type 2 diabetes in the STOP-NIDDM trial. *Diabetologia*.

[74] Liang H., Ward W. F. (2006). PGC-1*α*: a key regulator of energy metabolism. *American Journal of Physiology: Advances in Physiology Education*.

